# Association between leukocyte telomere shortening and exposure to traffic pollution: a cross-sectional study on traffic officers and indoor office workers

**DOI:** 10.1186/1476-069X-8-41

**Published:** 2009-09-21

**Authors:** Mirjam Hoxha, Laura Dioni, Matteo Bonzini, Angela Cecilia Pesatori, Silvia Fustinoni, Domenico Cavallo, Michele Carugno, Benedetta Albetti, Barbara Marinelli, Joel Schwartz, Pier Alberto Bertazzi, Andrea Baccarelli

**Affiliations:** 1 Center of Molecular and Genetic Epidemiology, Department of Preventive Medicine, IRCCS Maggiore Hospital, Mangiagalli and Regina Elena Foundation, Milan, Italy; 2 Department of Environmental and Occupational Health, Università degli Studi di Milano, Milan, Italy; 3 Department of Clinical and Biological Sciences, University of Insubria, Varese, Italy; 4 Epidemiology Unit, Department of Preventive Medicine, IRCCS Maggiore Hospital, Mangiagalli and Regina Elena Foundation, Milan, Italy; 5 Toxicology Unit, Department of Environmental and Occupational Health, IRCCS Maggiore Hospital, Mangiagalli and Regina Elena Foundation, Milan, Italy; 6 Department of Chemistry and Environmental Sciences, University of Insubria, Como, Italy; 7 Exposure, Epidemiology and Risk Program, Department of Environmental Health, Harvard School of Public Health, 401 Park Drive, Landmark Center, Suite 415 West, Boston, MA 02215, USA

## Abstract

**Background:**

Telomere shortening in blood leukocytes has been associated with increased morbidity and death from cardiovascular disease and cancer, but determinants of shortened telomeres, a molecular feature of biological aging, are still largely unidentified. Traffic pollution has been linked with both cardiovascular and cancer risks, particularly in older subjects. Whether exposure to traffic pollution is associated with telomere shortening has never been evaluated.

**Methods:**

We measured leukocyte telomere length (LTL) by real-time PCR in blood DNA from 77 traffic officers exposed to high levels of traffic pollutants and 57 office workers (referents). Airborne benzene and toluene, as tracers for traffic exposure, were measured using personal passive samplers and gas-chromatography/flame-ionization detector analysis. We used covariate-adjusted multivariable models to test the effects of the exposure on LTL and obtain adjusted LTL means and 95% Confidence Intervals (CIs).

**Results:**

Adjusted mean LTL was 1.10 (95%CI 1.04-1.16) in traffic officers and 1.27 in referents (95%CI 1.20-1.35) [p < 0.001]. LTL decreased in association with age in both traffic officers (p = 0.01) and referents (p = 0.001), but traffic officers had shorter LTL within each age category. Among traffic officers, adjusted mean relative LTL was shorter in individuals working in high (n = 45, LTL = 1.02, 95%CI 0.96-1.09) compared to low traffic intensity (n = 32, LTL = 1.22, 95%CI 1.13-1.31) [p < 0.001]. In the entire study population, LTL decreased with increasing levels of personal exposure to benzene (p = 0.004) and toluene (p = 0.008).

**Conclusion:**

Our results indicate that leukocyte telomere length is shortened in subjects exposed to traffic pollution, suggesting evidence of early biological aging and disease risk.

## Background

Telomeres are repetitive sequence nucleotides (TTAGGG)n positioned at the end of chromosomes that protect extremities from nucleolytic degradation and maintain chromosomal structural integrity [1]. Telomeres are widely regarded as the internal biological clock of a living organism, as their length shortens with age in all replicating somatic cells that have been examined [2-5]. Critically short telomeres are assumed to have functional implications, such as the induction of cellular senescence, which is characterized by the expression of specific markers of aging and the inability of the cell to divide further [2]. Features of biological aging vary considerably among individuals, and such differences may reflect a variety of environmental factors that affect oxidative stress and inflammation and, consequently, accelerate telomere shortening [2,6,7]. In human studies, telomere length measured in leukocyte DNA decreases with age [8,9], and telomere attrition in blood leukocytes has been shown to be accelerated by environmental factors that enhance biological aging, such as smoking [4] and oxidative stress [10]. Epidemiological investigations have demonstrated that individuals with shorter telomere length in circulating leukocytes have decreased life-expectancy [8,9,11], and increased risk of myocardial infarction [12,13], severe coronary heart disease [14], heart failure [15], hypertension [16], stroke [13], and cancer [17].

Pollution from traffic emissions, a major contributor of air pollutant exposure in urban areas, has been related to premature morbidity and mortality from cardiovascular disease and cancer in several epidemiological studies [18-21]. Exposure to traffic pollutants has been linked with activation of biological processes, such as production of Reactive Oxygen Species (ROS) [22,23] and activation of inflammatory pathways [24,25], which have been related to accelerated telomere shortening [2,4].

Whether exposure to traffic pollution is related with telomere shortening has never been determined. In the present work, we evaluated the effects of traffic pollution on telomere length measured in blood leukocytes of healthy subjects with different levels of exposure to traffic.

## Methods

### Study Participants and Exposure Assessment

The study included 77 street traffic officers and 57 office workers (referents) examined in Milan, Italy, between October 1999 and June 2000 (Table 1). At the time of examination, all study participants had been employed for at least one year. The traffic officers were assigned to full time traffic duties in inner-city Milan (7-hour shifts from 6:30 am to 1:30 pm), and did not include motorcycling or car patrol officers. Individual written informed consent was obtained from all participants before the study. The study was approved by the Institutional Review Board of the Maggiore Hospital Foundation, Milan, Italy. A structured questionnaire was used to collect information on lifestyle, and risk factors. Traffic officers were also asked to evaluate the usual traffic conditions during their work shifts by a specific questionnaire item that gave two possible choices (low traffic or high traffic). None of the subjects had acute inflammatory conditions or other concurrent illnesses, as shown by responses to specific questionnaire items, as well as by no major abnormalities in differential blood counts.

**Table 1:** General characteristics of the study subjects

	Referents	Traffic officers	**p-value**^**a**^
	(n = 57)	(n = 77)	
Age, n (%)			

<30 years	16 (28%)	36 (47%)	

30-40 years	19 (33%)	31 (40%)	

>40 years	22 (39%)	10 (13%)	0.002

Gender, n (%)			

Male	38 (67%)	47 (61%)	

Female	19 (33%)	30 (39%)	0.58

Cigarette smoking, n (%)			

Never	26 (46%)	40 (52%)	

Ever	31 (54%)	37 (48%)	0.49

Cigarettes/day, n (%)^b^			

1-10 cigarettes/day	9 (40%)	5 (18%)	

11-20 cigarettes/day	7 (30%)	16 (57%)	

>20 cigarettes/day	7 (30%)	7 (25%)	0.15

Pack-years of smoking, n (%)			

0 pack-years	26 (45%)	40 (52%)	

0.1-10 pack-years	14 (25%)	22 (28%)	

>10 pack-years	17 (30%)	15 (20%)	0.39

Exposure to environmental tobacco smoke, n (%)^c^			

No	14 (54%)	24 (60%)	

Yes	12 (46%)	16 (40%)	0.30

Alcohol consumption, n (%)^d^			

Occasional/never	24 (44%)	40 (56%)	

Every week	14 (26%)	18 (26%)	

Every day	16 (30%)	13 (18%)	0.27

^a^Fisher exact test for differences across categories

^b^Data for current smokers are shown

^c^Data for never smokers are shown

^d^Subject count not add up to the total number of participants due to missing values

Airborne benzene and toluene, taken as tracers of traffic exposure [26], were measured by a passive sampler (stainless steel tube, 9 mm diameter × 90 mm length; containing Chromosorb 106) worn near the breathing zone by each participant for one entire work shift. Passive sampling allows to obtain an integrated measure of the airborne pollutant levels over the entire time period the sampler is worn. Benzene and toluene concentrations were determined by thermal desorption followed by gas chromatography/flame-ionization detector analysis. None of the study subjects was exposed during the monitoring period to sources of benzene or toluene other than traffic pollution such as paints, glues, adhesives, varnishes, lacquers, or shoe polishes. The day following work shift in which airborne benzene and toluene exposure was assessed, a 7 ml whole blood sample was collected in EDTA tube. All samples were collected at 8:30 AM.

### Telomere length measurement

DNA was extracted from whole blood using the Nucleon™ BACC2 genomic DNA extraction kit (Amersham). Leukocyte Telomere Length (LTL) was measured in blood genomic DNA using the quantitative real-time method described by Cawthon [27], which measures the relative LTL by determining the ratio of telomere repeat copy number (T) to single copy gene (S) copy number (T/S ratio) in experimental samples relative to a reference sample. DNA samples from referents and traffic officers were interspersed across PCR plates.

The T (telomere) PCR mix was: iQ SYBR Green Supermix (Bio-Rad) 1×, tel1b 100 nM, tel2b 900 nM, DMSO 1%, EDTA 1×. The S (human beta-globin) PCR mix was: iQ SYBR Green Supermix (Bio-Rad) 1×, hbg1 300 nM, hbg2 700 nM, DMSO 1%, DTT 2,5 mM, EDTA 1×. We used the PCR primer sets previously described by Mc Grath et al. [28]. We used pooled DNA from 20 referents (500 ng for each sample), randomly selected from samples of this same study, to create a fresh standard curve, ranging from 8 ng/μl to 0,5 ng/μl, at every T and S PCR run. All samples contained E. coli DNA heated at 96°C × 10 minutes and cooled at room temperature. 15 ng of DNA samples was added to each reaction (final volume 20 μl). All PCRs were performed on a DNA Engine thermal cycler Chromo4 (Bio-Rad, Hercules, California, USA). The thermal cycling profile for both amplicons started with a 95°C incubation for 3 minutes to activate the hot-start iTaq DNA polymerase. The T PCR continued with 25 cycles at 95°C for 15s, and anneal/extend at 54°C for 49s. The S PCR continued with 35 cycles at 95°C for 15s, anneal at 58°C for 1s, extend at 72°C for 15s. At the end of each reaction, a melting curve was used for both T and S PCRs. All samples were run in triplicates and the mean of three measurements was used in the statistical analyses.

### Statistical Analysis

Telomere length was log-transformed to approximate normal distribution. Consequently, we present telomere length data as geometric means and 95% Confidence Intervals (CIs). In univariate analysis, we used the Fisher's exact test to evaluate the association of telomere length with individual characteristics, which were all presented as categorical variables. As traffic officers and referents differed in their age distribution, we tested for differences in telomere length between the two groups using regression models that adjusted for age (fitted as a continuous variable), in addition to gender, and smoking (ever/never), and pack years of smoking. We used multivariable regression models adjusting for the same variables (fitted as a continuous variable) also to evaluate the association of benzene or toluene with telomere length. As a sensitivity analysis, we fitted a set of models that included a quadratic term for age in addition to the linear term. Result from this set of models showed only marginal differences from the results reported in the paper. All statistical tests were two-sided. A p-value < 0.05 was considered statistically significant. All analyses were performed in Stata 9.0 (Stata Corp., College Station, TX).

## Results

### Characteristics of the Study Population and Personal Exposure to Airborne Traffic Pollutants

The characteristics of the study subjects are shown in Table 1. Traffic officers were moderately but significantly younger than referents (p = 0.002). The two groups were otherwise similar for the other characteristics that we considered. Mean airborne benzene levels, measured by personal breathing zone samplers during the entire work shift, were 13.0 μg/m^3 ^(95% CI 8.2-17.9) in referents and 31.8 μg/m^3 ^(95% CI 22.6-40.9) in traffic officers (Table 2). Mean airborne toluene levels were 43.4 μg/m^3 ^(95% CI 30.5-56.2) in referents and 128.7 μg/m^3 ^(95% CI 73.5-183.9) in traffic officers (Table 2). Airborne benzene and toluene levels were highly correlated in this study population (r^2 ^= 0.87). Among traffic officers, 32 individuals reported to be usually exposed to low traffic intensity, and 45 to high traffic intensity. Individuals reporting exposure to low traffic intensity had mean airborne benzene of 26.3 μg/m^3 ^(95% CI 19.9-32.6) and mean airborne toluene of 101.1 μg/m^3 ^(95% CI 66.8-137.0), whereas individuals reporting exposure to high traffic intensity had mean airborne benzene of 35.7 μg/m^3 ^(95% CI 20.6-50.8) and mean airborne toluene of 147.8 μg/m^3 ^(95% CI 55.3-240.8).

**Table 2:** Personal exposure level^a ^to airborne benzene and toluene in indoor office workers (referents) and traffic officers

	Referents (n = 57)			Traffic officers (n = 77)		
	**Mean**	**(95% CI)**	**Min/Max**	**Mean**	**(95% CI)**	**Min/Max**

Benzene, μg/m^3^	13.0	(8.2-17.9)	2.0/115.1	31.8	(22.6-40.9)	9.0/315.7
Toluene, μg/m^3^	43.4	(30.5-56.2)	6.0/368.0	128.7	(73.5-183.9)	24.4/1710.7

^a^Airborne benzene and toluene concentrations measured as a tracer of traffic exposure measured through a passive sampler worn near the breathing zone by each participant during work shift. Airborne benzene and toluene levels showed high correlation in this study population (r^2 ^= 0.87)

### Telomere length and individual characteristics

LTL decreased significantly in association with age in both referents (p = 0.001) and traffic officers (p = 0.01) [Table 3]. In each age category, traffic officers exhibited shorter telomeres than referents. Gender was not associated with LTL either in referents or traffic officers. Among referents, LTL was shorter in ever smokers (p = 0.04), particularly in those with a higher number of pack-years of smoking (p = 0.05). The relationship between smoking habits (ever/never) and telomere length was borderline significant after adjusting by age (p = 0.06), while the effect of pack-years was not statistically significant (p = 0.16) [Table 3]. Among traffic officers, smoking and pack-years were not statistically associated with LTL (Table 3). Mean relative LTL was not associated with number of cigarettes/day, exposure to environmental tobacco smoke, or alcohol consumption, either in referents or traffic officers (Table 3). In addition, we evaluated whether LTL was associated with results from complete blood counts. LTL was not associated in traffic officers or referents with white blood cell count, or proportions of neutrophils, lymphocytes, monocytes, basophils, and eosinophils (data not shown).

**Table 3:** Telomere length by individual characteristics of indoor office workers (referents) and traffic officers

	Referents (n = 57)					Traffic officers (n = 77)				
	**n**	**mean**^**a**^	**(95% CI)**^**a**^	**p-value**^**b**^	**p-value**^**c**^	**n**	**mean**^**a**^	**(95% CI)**^**a**^	**p-value**^**b**^	**p-value**^**c**^

Age										

<30 year	16	1.44	(1.31-1.59)			36	1.22	(1.11-1.33)		

30-40 year	19	1.21	(1.09-1.33)			31	1.04	(0.98- 1.11)		

>40 year	22	1.14	(1.03-1.26)	0.001	-	10	1.03	(0.91-1.17)	0.01	-

Gender										

Male	38	1.24	(1.15-1.34)			47	1.12	(1.04-1.20)		

Female	19	1.23	(1.10-1.38)	0.90	0.74	30	1.12	(1.04-1.22)	0.91	0.71

Cigarette smoking										

Never smokers	26	1.33	(1.20-1.48)			40	1.16	(1.07-1.26)		

Ever smokers	31	1.17	(1.10-1.25)	0.04	0.06	37	1.08	(1.01-1.15)	0.17	0.33

Cigarettes/day^d^										

1-10 cig./day	9	1.19	(1.03-1.37)			5	1.27	(0.94-1.73)		

11-20 cig./day	7	1.20	(1.05-1.38)			16	1.05	(0.96-1.14)		

>20 cig./day	7	1.19	(0.96-1.47)	0.99	0.47	7	1.09	(0.98-1.22)	0.22	0.38

Pack-years										

0 pack-years	26	1.33	(1.20-1.48)			40	1.15	(1.06-1.25)		

0.1-10 pack-years	14	1.19	(1.07-1.32)			22	1.11	(1.00-1.23)		

>10 pack-years	17	1.16	(1.05-1.28)	0.05	0.16	15	1.05	(0.97-1.14)	0.18	0.43

Exposure to environmental tobacco smoke^e^										

No	14	1.27	(1.10-1.46)			24	1.18	(1.06-1.31)		

Yes	12	1.40	(1.17-1.68)	0.26	0.39	16	1.12	(0.98-1.30)	0.59	0.31

Alcohol consumption, n(%)^f^										

Occasional/never	24	1.26	(1.15-1.37)			40	1.20	(1.11-1.28)		

Every week	14	1.28	(1.16-1.39)			18	1.15	(0.96-1.35)		

Every day	16	1.24	(1.04-1.46)	0.93	0.27	13	1.05	(0.97-1.13)	0.13	0.41

^a^Statistical analysis on telomere length were performed on log-transformed data to approximate normal distribution. Geometric means and 95% Confidence Intervals (CIs) are reported

^b^p-values from t-test (two-class variables) or regression-based test for trend (variables with three categories) for differences in telomere length by subjects' characteristics

^c^Age adjusted p-values from regression-based test for trend for differences in telomere length by subjects characteristics. Age was fitted as a continuous variable in the models.

^d^Data for current smokers are shown

^e^Data for never smokers are shown

^f^Subject count not add up to the total number of participants due to missing values

### Telomere length and traffic exposure

**Figure 1:**
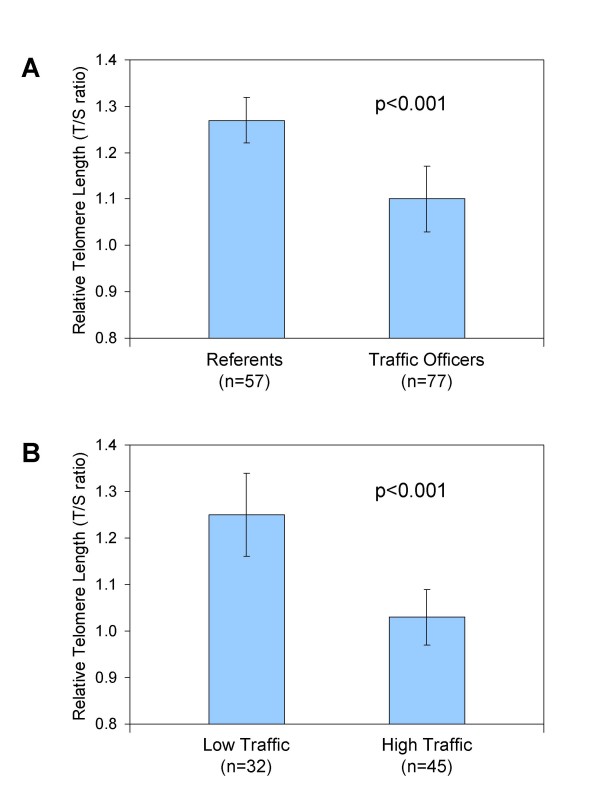
**Differences in leukocyte telomere length between (A) indoor office workers (referents) and traffic officers; and (B) traffic officers with exposure to low and high traffic intensity during their work shift**. The graphs show geometric means (bars) 95% confidence intervals (lines) of telomere length adjusted by age, gender, smoking (ever/never), and pack-years.

Mean relative LTL, estimated in models adjusting for age, gender, smoking (ever/never), and pack-years, was shorter in traffic officers (mean = 1.10, 95% CI 1.04-1.16) than in referents (mean = 1.27, 95% CI 1.20-1.35) [p < 0.001, Figure 1A]. Among traffic officers [Figure 1B], relative LTL was shorter in individuals working in high traffic intensity (mean = 1.02, 95% CI 0.96-1.09) compared to those working in low traffic intensity (mean = 1.22, 95% CI 1.13-1.31) [p < 0.001]. LTL in traffic officers working in low traffic intensity was not significantly shorter than in referents.

We found no association between duration of employment and LTL. Among traffic officers, individuals with shorter duration of employment (1-2 years, n = 48) had adjusted mean LTL of 1.09 (95% CI 1.01-1.17), whereas individuals with longer employment (3-17 years) had adjusted mean LTL of 1.11 (95% CI 1.03-1.21) [p = 0.62].

### Telomere length and levels of traffic pollutants

**Figure 2:**
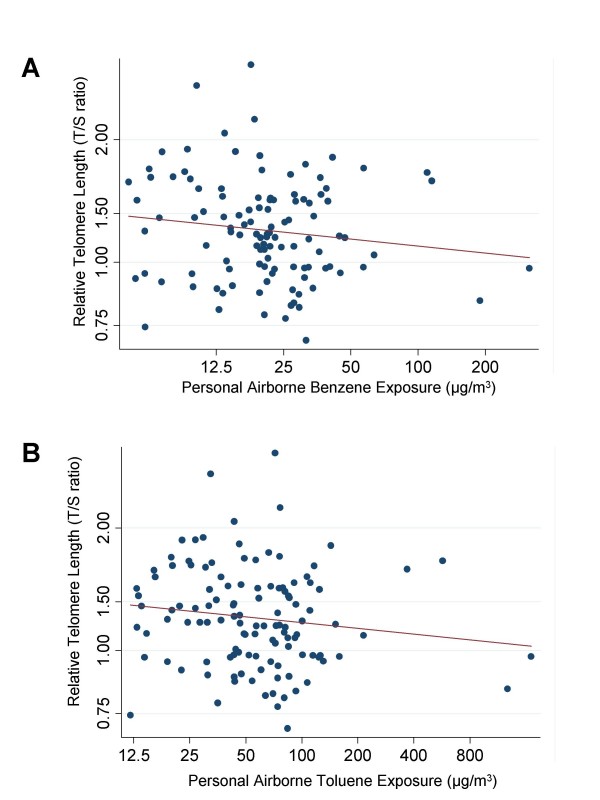
**Decline of leukocyte telomere length associated with increasing levels of personal airborne of (A) benzene (p = 0.004) and (B) toluene (p = 0.008)**.

LTL decreased in association with increasing personal levels of airborne benzene (Figure 2A) and toluene (Figure 2B). In models adjusting for age, gender, smoking (ever/never), and pack-years, we estimated that an increase in airborne benzene exposure level equal to the difference between the 25^th ^and 75^th ^centile (interquartile difference = 11.2 μg/m^3^) was associated with a 6.4% (95% CI, 2.1%-10.4%) decrease in LTL (p = 0.004). An increase in airborne toluene exposure level equal to the difference between the 25^th ^and 75^th ^centile (interquartile difference = 25.7 μg/m^3^) was associated with a 6.2% (95% CI 1.7%-10.4%) decrease in LTL (p = 0.008). To get a perception of the magnitude of benzene and toluene effects, LTL decreases associated with the exposures can be compared to the percent decrease in LTL associated with age. Among the referent subjects, each year of age was associated with a 0.5% (95% CI 0.1%-0.9%) decrease in LTL. As a sensitivity analysis, we tested for the association of benzene and toluene with LTL after excluding the two study participants with the highest levels of exposure. In this analysis, the effects of benzene and toluene remained statistically significant (6.1%, 95% CI 1.5%-10.4%, p = 0.01 for benzene, and 6.0%, 95% CI 1.0%-10.7%, p = 0.02 for toluene).

## Discussion

Our findings indicate that exposure to widespread environmental pollutants, such as those from traffic emissions, may contribute to determine early biological aging, as chronicled by leukocyte telomere length. Traffic emissions are a mix of by-products of the combustion process including hundreds of pollutants in gaseous and particulate phases. Particles may stimulate directly the generation of ROS, or through transition metal and quinone structures that undergo redox cycling [29]. Other combustion products, including nitric oxide and benzene, have been shown to generate oxidative stress [30-32]. In addition, exposure to traffic pollutants may indirectly produce oxidative stress by activation of inflammatory cells capable of generating ROS and reactive nitrogen species [29]. Oxidative stress and inflammation, which have been shown to increase the rate of leukocyte telomere attrition [2-4,7,33], are widely considered as two main mediators of the effects of traffic pollutants on human health [22-24]. Oxidative stress can shorten telomeres in vitro, and antioxidants decrease shortening [34]. Due to their specific sequence with high guanine content, telomeres are remarkably sensitive to damage by oxidative stress [33]. In addition, telomeric DNA is deficient in the repair of single-strand breaks, which are induced by ROS either directly or as an intermediate step in the repair of oxidative base modifications [2]. Inflammatory processes cause heightened leukocyte turnover rate that can result in a greater loss of leukocyte telomere repeats over time [2,4,33].

In our results, exposure to traffic appeared to anticipate the age-related decrease in telomere length. In particular, albeit telomere length was associated with age in both traffic officers and referents, when leukocyte telomere length was stratified by age (Table 3), traffic officers exhibited lengths that were similar to those observed in referents roughly ten years older. Similarly, an interquartile range change in quantitatively measured airborne benzene and toluene exposure was associated with a 6.4% and 6.2% decrease in LTL respectively, which was similar to that associated with a difference of 13 years in age, considering that a 0.5% decrease per each year of age in LTL was found in our study. Our data are based on a cross-sectional study, which does not provide direct information on dynamic changes in LTL over time. In addition our study did not have measures of long-term exposure to traffic pollutants. Future studies should prospectively address whether LTL decreases more rapidly in traffic exposed subjects as their age.

In addition, we found that smoking decreased blood telomere length, consistently with previous results [4]. In our study, ever smoking was associated with shorter telomeres among referent subjects, but we did not find an association with pack-years of smoking, cigarettes number, and exposure to environmental tobacco smoke possibly due to limited statistical power to test for such associations in our study. Also, the association between ever smoking and LTL was only borderline significant after adjustment by age.

Blood leukocytes have been used in several studies of telomere length that have related telomere shortening with smoking [4], as well as with myocardial infarction [12,13], severe coronary heart disease [14], heart failure [15], hypertension [16], stroke [13], cancer [17], and decreased life-expectancy [8,9,11]. Rates of telomere shortening have been shown to be similar in different tissues, thus easily accessible tissues such as blood leukocytes may serve as surrogates for telomere length in tissues involved in age-related diseases [33].

The mechanisms linking shorter LTL with disease risk still need to be fully apprehended. Telomere shortening is universally accepted as a marker of individual and cellular aging [5,7]. Cellular senescence is a major feature of atherosclerotic plaques [35]. In in-vitro models, telomere shortening in coronary endothelial cells has been associated with expression of molecules implicated in atherogenesis [36]. Short telomeres have been also associated with increased genetic instability. Shortened telomeres lose their capping function at the end of chromosomes. Such dysfunctional telomeres can result in sister cromatid fusion and breakage/fusion/bridge leading to non reciprocal translocation and chromosomal rearrangement, commonly associated with human cancer [37].

Our study was based on a sample of traffic officers and indoor office workers with different degrees of exposure to traffic pollutants. We found shortened LTL in traffic officers, particularly in those working in high traffic conditions. In addition, we further characterized the exposure of the study participants using personal measures of airborne benzene and toluene, taken as tracers of traffic emissions [38]. In an Italian study conducted in the same years as our study that also included traffic officers [26], personal benzene exposure was shown be more tightly correlated with local traffic intensity than other measures of air pollution such as particulate matter of aerodynamic diameter <10 μm (PM_10_) measured by personal monitors.

In our study, the referent subjects were moderately older than the traffic officers. To account for this inequality, which would have decreased the observed difference in telomere length, we used multivariable analyses that adjusted our results by age, as well as by other potential confounders, including gender, smoking, and pack-years.

Our results, coupled with previous evidence showing that telomere length is associated with increased incidence of cardiovascular disease and cancer [12-17], lead us to speculate that shortened telomeres are an intermediate step between exposure to traffic pollutants and health-related effects. However, we cannot exclude that telomere length may just reflect (and thus serve as useful indicators of) the biological history of cells and individuals [8], without a mechanistic role in the development of exposure-related health outcomes. In addition, exposure to traffic noise might have contributed to determine shorter LTL in our exposed group. Noise exposure has also been shown to increase oxidative stress [39]. Because we did not collect any information on noise exposure in our study, we were unable to test whether traffic noise determined shorter LTL in our exposed group. Cherkas et al. [40] have found that LTL was longer in subjects who reported higher leisure-time physical activity. However, Cherkas et al. suggested that intermittent physical activity specifically in leisure-time has beneficial effects on LTL, because adjusting LTL for physical activity at work did not affect their results. In our study, we found shorter LTL in the exposed group was compared to a group of office worker referents, who might have more sedentary work. Although we did not collect information on work-related and leisure-time physical activities, we expect that differences in work-related physical activities between the two exposure groups might have decreased, rather than caused, the differences in LTL we found in our study.

## Conclusion

We showed that leukocyte telomere length is shortened in subjects exposed to traffic pollution, particularly in subjects exposed to intense traffic and with higher levels of personal exposure to airborne traffic pollutants. Our findings indicate that early biological aging, as reflected in telomere shortening, may mediate the effects of traffic exposure on human health.

## Abbreviations

LTL: Leukocyte Telomere Length; 95% CI: 95% Confidence Interval; ROS: Reactive Oxygen Species.

## Competing interests

The authors declare that they have no competing interests.

## Authors' contributions

ACP, JS, PAB, and AB designed research. MH, LD, SF, DC, MC, BA, BM performed research. MB analyzed data. MH and AB wrote the paper. All authors read and approved the final manuscript.
